# Polygenic Risk Score and Rare Variant Burden Identified by Targeted Sequencing in a Group of Patients with Pigment Epithelial Detachment in Age-Related Macular Degeneration

**DOI:** 10.3390/genes14091707

**Published:** 2023-08-27

**Authors:** Anna Wąsowska, Adam Sendecki, Anna Boguszewska-Chachulska, Sławomir Teper

**Affiliations:** 1 Chair and Clinical Department of Ophthalmology, Faculty of Medical Sciences in Zabrze, Medical University of Silesia, 40-055 Katowice, Poland; 2 Genomed S.A., 02-972 Warszawa, Poland

**Keywords:** pigment epithelial detachment, age-related macular degeneration, PED susceptibility, AMD genetics, polygenic model, polygenic risk score

## Abstract

A subset of ophthalmic imaging examination results from 334 patients were subjected to reanalysis to identify a specific group of patients with pigment epithelial detachment (PED) in at least one eye. Overall, we found a subgroup of 47 patients manifesting PED and studied their genotypes in comparison to those of patients with age-related macular degeneration without PED and healthy controls. We established a polygenic risk score that allowed the explanation of 16.3% of the variation within the disease. The highest predictive value was achieved for a model consisting of six non-coding variants: rs760306 (*BEST1*), rs148662546 (*BEST1*), rs11569560 (*C3*), rs74600252 (*GUCA1B*), rs2240688 (*PROM1*), and rs185507582 (*TCF4*). The risk of PED occurrence was found to be the highest in the first tercile, showing a 7.89-fold higher risk compared to the third tercile for AMD without PED (95% CI: 2.87; 21.71, *p* < 0.001) and a 7.22-fold higher risk compared to the healthy controls (95% CI: 2.60; 20.06, *p* < 0.001). In addition, we focused on rare variants in targeted genes. The rare variants’ burden was compared among the groups, but no statistical significance was observed in the number of rare variants, predicted functional effects, or pathogenicity classification.

## 1. Introduction

Age-related macular degeneration (AMD) is a complex multifactorial disease that affects the macula, the central part of the retina. It is the leading cause of irreversible vision loss in individuals over the age of 50 years in developed countries [1,2]. AMD is a progressive disorder characterised by the formation of drusen, the accumulation of extracellular deposits beneath the retinal pigment epithelium (RPE), and the development of geographic atrophy (GA) or choroidal neovascularisation (CNV) [3]. One of the complications that can arise during disease progression is the detachment of the pigment epithelium (PED), which further exacerbates the visual impairment and functional deficits in affected individuals.

Pigment epithelial detachment refers to the separation or detachment of the RPE layer from the underlying Bruch’s membrane, resulting in functional and structural changes that contribute to visual impairment. It is a clinical finding that can occur in various retinal disorders, including both types of AMD. There are three primary types of PED: serous, fibrovascular, and drusenoid PED. Serous PED is characterised by the accumulation of fluid between the RPE and Bruch’s membrane, resulting in a blister-like elevation of the RPE layer. Fibrovascular PED, on the other hand, is associated with the growth of abnormal blood vessels beneath the RPE, leading to the detachment of the RPE layer. The latter, drusenoid PED, occurs as a result of the drusen merging together, and it is linked to the advancement of GA and the progression to neovascular age-related macular degeneration, as well as the formation of calcified drusen and alterations in pigmentation [4].

Neovascular AMD (nAMD) associated with PED poses an additional risk of RPE tearing and rapid deterioration of visual acuity. This risk is increased by treatment with VEGF inhibitors, the first-line drugs for this disease. This complication is probably the result of shrinkage of the neovascular tissue often located at the edge of the PED [5]. It should also be noted that the treatment of nAMD with PED is considered challenging and often requires intensive therapy, and the effects are sometimes complicated by the development of atrophy [6].

Numerous risk factors have been identified for AMD and PED, including age [7], genetic predisposition, smoking, and others. The contribution of genetic factors to macular degeneration has been widely investigated. Genome-wide association studies (GWASs) have identified numerous common genetic variants associated with AMD, providing valuable insights into the disease’s pathogenesis. These studies have highlighted the involvement of genes related to various biological processes, including complement activation, lipid metabolism, extracellular matrix remodelling, and angiogenesis [3,8,9]. However, the cumulative effect of these variants on an individual’s risk of developing AMD and PED remains to be fully elucidated.

The overall risk of developing a complex disease may be influenced by the combined effects of multiple common genetic variants in various genes. Each variant individually has a relatively small or moderate impact on the overall risk of the disease. Polygenic risk scores (PRSs) have emerged as a promising approach to estimating an individual’s genetic susceptibility to complex diseases, including AMD and PED. A PRS is calculated by summing the weighted contributions of multiple genetic variants based on their effect sizes obtained from large-scale GWASs of a related trait that shares a common genetic basis with the disease, along with genotyping data from a specific group of patients, such as targeted sequencing. By aggregating the effects of multiple genetic variants, PRSs provide a more comprehensive assessment of an individual’s genetic risk profile.

The application of PRSs in case/control studies of AMD with PED holds great potential. It allows for the evaluation of the cumulative genetic risk burden in a specific phenotype, shedding light on the underlying genetic architecture and enabling risk stratification [10]. Several studies have demonstrated the utility of polygenic predictive models in stratification of the risk of AMD and related complications, such as CNV. For example, Hageman et al. established a polygenic risk score for CNV using 13 AMD-associated variants in eight loci [11], while Grassmann et al. developed an eight-locus risk assessment model associated with predicting AMD [12].

While PRSs primarily capture the contributions of common genetic variants, the role of rare variants in AMD and PED should not be overlooked. Rare variants with larger effect sizes can contribute significantly to the disease risk in specific individuals or families, as shown by Lu et al. [13,14]. Incorporating rare variant analysis alongside a PRS can provide a more comprehensive understanding of the genetic landscape of AMD and PED. Recent studies have highlighted the relevance of rare variants in AMD, such as those in the *CFH* and *C3* genes, that have been associated with increased risk of developing AMD [3].

In this study, we aimed to analyse the genetic background of AMD with PED, which represents a distinct phenotype within the spectrum of AMD, among Polish patients. By conducting a comprehensive genetic analysis, including a GWAS and targeted sequencing approaches, we hope to identify the genetic risk score for common variants, as well as rare variants, associated with the risk of PED in AMD in the Polish population.

In addition to the evaluation of the polygenic risk score, we also assessed the genomic burden of rare variants within gene coding sequences and compared it among the tested groups (AMD with PED, AMD without PED, controls).

By elucidating the genetic determinants of PED in AMD among Polish patients, this study aims to contribute to the understanding of the pathogenesis of PED and provide insights into personalised approaches for risk assessment, early detection, and potential therapeutic interventions in this population.

## 2. Materials and Methods

### 2.1. Sample Data

Data were obtained from the NeuStemGen STRATEGMED project that was conducted in years 2014–2019 by the consortium of the Chair and Clinical Department of Ophthalmology, Faculty of Medical Science, Medical University of Silesia in Katowice, the First Department of Ophthalmology, Pomeranian Medical University in Szczecin, and Genomed S.A. company (Warszawa, Poland); this was approved by the Ethics Committee of the Medical University of Silesia (Resolution No KNW/0022/KB1/105/13) and followed the tenets of The Declaration of Helsinki. A subset of ophthalmic imaging examination results from 334 patients recruited by the Chair and Clinical Department of Ophthalmology, Faculty of Medical Sciences in Zabrze, Medical University of Silesia in Katowice was subjected to reanalysis. The aim was to identify a specific group of patients with pigment epithelial detachment in at least one eye. The reanalysis focused on digital fundus images, cross-section macular OCT (using radial and 3D wide-scanning protocols), and OCT angiography (OCT-A) images. Overall, 47 patients manifested PED in at least one eye.

### 2.2. PRS Calculation

The GWAS summary statistics utilised to establish the polygenic risk score in relation to age-related macular degeneration were derived from the GERA European ancestry cohort study. These summary statistics, which served as the base data for the PRS, were published by Guindo-Martinez. et al. [7] and are available in the NHGRI-EBI GWAS Catalog (study accession GCST90086108). In our study, we chose the additive model, which aligned with a PRS model that was previously described for the development of AMD in a cohort of Polish patients [14]. One of our objectives was to assess the correlation between both PRS models; hence the consistent choice of the additive model in our analysis. The NeuStemGen STRATEGMED project aimed to genotype a cohort of Polish patients with control samples with custom 30-gene panel targeted sequencing using molecular inverted probes and next-generation sequencing on an Illumina platform. The details of library preparation and sequencing were described previously by Ulanczyk et al. [15], and the data analysis, including mapping to the reference genome, variant detection, and filtering, was described in our previous publication [16]. Variants determined by targeted sequencing were subjected to QC analysis, which was performed using PLINK v1.90 [17], following data quality control protocols suggested by Anderson et al. [18] and Clarke et al. [19]. At the filtering step, individuals with genotype gaps exceeding 10% were excluded from the study, as were variants with a genotyping rate below 5%. Additionally, rare single-nucleotide polymorphisms (SNPs) with a minor allele frequency (MAF) lower than 0.5% and variants that deviated from the Hardy–Weinberg equilibrium (with *p* < 1 × 10^−5^) were also filtered out.

In the first step, the variants remaining after filtering were used to perform association tests within all of the AMD patients using Cochrane–Armitage trend test to detect SNPs that distinguished AMD patients with PED and those without PED. All of the variants with *p*-value < 0.05 were then subjected to PRSice2 [20] for polygenic risk score estimation with the “Clumping and thresholding” method. The parameters were set to a 100 kb window size, 0.5 squared correlation threshold r^2^, and default *p*-value threshold to retain the most effective variant within the locus according to linkage disequilibrium and the *p*-value. The additive model was used, followed by 10,000 permutations, to avoid model overfitting and achieve the empirical *p*-value. The established model was then used to calculate the PRS values for the healthy controls. The association between the achieved PRS and the target trait was tested using logistic regression, and it was measured in terms of phenotypic variance explained (Nagelkerke’s pseudo-R2). The discriminative power in disease prediction was calculated using the area under the receiver operating curve (AUC). The statistical analysis and visualizations were performed in the R Software version 4.2.2 (The R Foundation for Statistical Computing, Vienna, Austria) with appropriate packages (dplyr [21], epitools [22], pROC [23], and ggplot2 [24], among others).

### 2.3. Rare Variant Evaluation

Due to the relatively small population size for rare variant discovery, we selected only regions that were well covered in all samples and considered variants observed in a maximum of two patients for heterozygous variants and a maximum of one patient for homozygous variants (MAF threshold of 0.3%). Furthermore, we cross-referenced each variant with the Genome Aggregation Database (gnomAD v2.1.1 [25]) to determine its allele frequency in the non-Finnish European population and in all populations.

To assess the functional effects of the variants, we utilised PolyPhen [26] for functional effect prediction. Additionally, we obtained information about the variants’ pathogenicity from the Varsome database (accessed on 9 June 2023 [27]) by using the germline classification calculated according to the recommendations of the American College of Medical Genetics and Genomics (ACMG) [28]. The ACMG classification system incorporates various criteria for evaluating the pathogenicity of genetic variants, including population frequency, computational predictions, functional studies, and segregation data, among others. The significance was tested using either the Fisher exact test or chi-square test.

## 3. Results

### 3.1. Sample Data

This observational study included a diverse group of 334 patients who presented to our ophthalmology clinic. Patients were diagnosed based on comprehensive ophthalmic examinations, including visual acuity assessment, fundus photography, optical coherence tomography, and angiography or angio-OCT. Pigment epithelial detachment (PED) is a common feature observed in patients with AMD, presenting as an elevation of the retinal pigment epithelium. One of the aims of this study was to evaluate the characteristics of PED in patients with AMD using multimodal swept-source OCT Triton imaging (Topcon). Diagnosis of AMD and associated PED subtypes was established based on the standardised grading criteria outlined by the Age-Related Eye Disease Study Group. Demographic information, clinical data, and imaging findings were collected and analysed. The clinical spectrum of AMD can vary widely, with subtypes such as serous, drusenoid, and fibrovascular pigment epithelial detachments (PEDs) further complicating its presentation. Understanding the demographics and characteristics of patients with AMD and associated pathologies is crucial for effective management and treatment strategies.

Among the 334 patients included in this study, 119 were male (35.6%) and 215 were female (64.4%). Of these patients, 190 were diagnosed with AMD, accounting for 56.9% of the cohort. Additionally, 47 patients presented with both AMD and PED, representing 14.1% of the cohort. Among the patients with AMD and PED, there were 31 cases of serous PED, accounting for 66% of the AMD–PED subgroup. Drusenoid PED was observed in 14 cases (29.8%), and fibrovascular PED was present in 2 cases (4.2%).

### 3.2. PRS Model

A total of 2348 variants genotyped for 334 patients were selected for further analysis and underwent quality control. Twelve variants were excluded from the analysis due to missing genotype data. Furthermore, 42 variants were found to deviate from the criteria of the Hardy–Weinberg equilibrium and were, therefore, removed from the analysis. Additionally, 1668 rare SNPs with a minor allele frequency below 0.5% were filtered out of the dataset. The remaining 626 variants were subjected to the Cochrane–Armitage test, resulting in a list of 28 variants localised in 13 genes with *p* < 0.05, which were then passed to PRSice2 to calculate the polygenic risk score model. The highest predictive value was achieved for a model consisting of six non-coding variants: rs760306 (*BEST1*), rs148662546 (*BEST1*), rs11569560 (*C3*), rs74600252 (*GUCA1B*), rs2240688 (*PROM1*), and rs185507582 (*TCF4*); these are presented in Table 1.

The PRS model’s variance explained was 16.3% with an empirical *p*-value of 9.9 × 10^−5^. To assess the quality of the estimated risk prediction model, a Hosmer–Lemeshow test was conducted, which indicated a good fit (χ^2^ = 6.6, *p* = 0.58). This test helped to determine how well the predicted risks aligned with the observed outcomes. Additionally, the examination of the diagnostic ability of the model was performed through receiver operating characteristic (ROC) analysis, which yielded an AUC of 0.75 (95% CI: 0.67–0.83) for AMD with PED versus AMD without PED and 0.72 (95% CI: 0.65–0.79) for AMD with PED compared to all samples without PED. The AUC represents the overall discriminatory power of the model, with higher values indicating better predictive performance. In this case, an AUC larger than 0.7 suggests a reasonably accurate predictive ability of the risk prediction model for the specific condition being evaluated.

**Figure 1:**
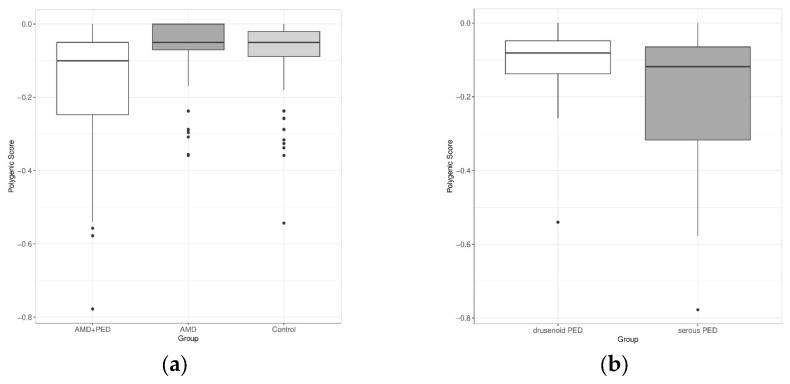
Boxplots showing (**a**) the PRS distribution in the tested groups and (**b**) the PRS distribution for the drusenoid and serous PED samples.

In Figure 1a, it can be observed that the overall median PRS for age-related macular degeneration with pigment epithelial detachments was lower by 0.041 compared to both AMD without PED and the control samples (−0.091 vs. −0.05, 95% CI: (−0.071; −0.038) and −0.091 vs. −0.05, 95% CI: (−0.068; −0.021), respectively). The statistical significance of this difference was confirmed using the Mann–Whitney test, with *p*-values of less than 0.001 for both comparisons. Moreover, the median PRSs for drusenoid and serous PED were compared (−0.081 vs. −0.118), but the difference did not reach statistical significance (Figure 1b). The samples with the fibrovascular PED were omitted due to the low sample size. Therefore, for further calculations, all PED types were treated as one group. 

**Figure 2:**
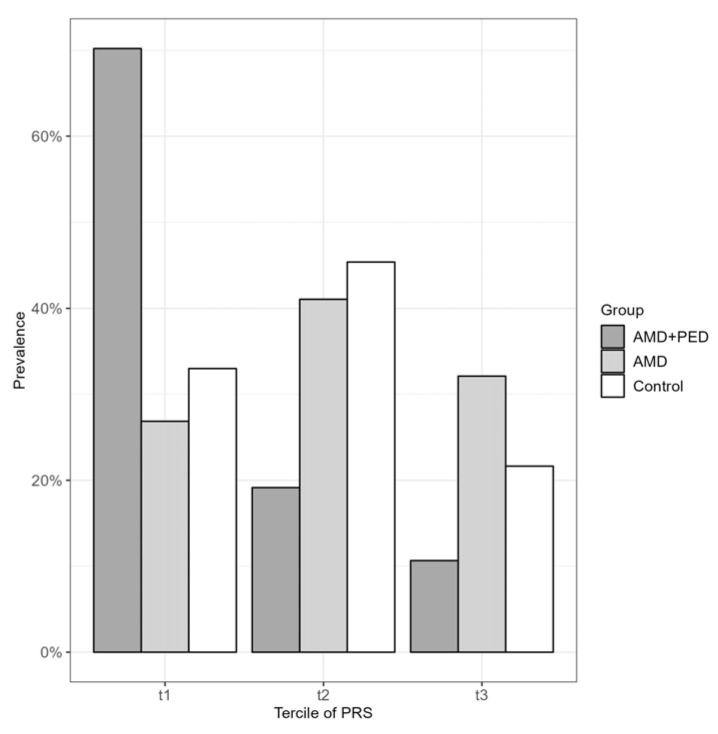
Prevalence of AMD with PED, AMD without PED, and controls in terciles of the polygenic risk score.

Furthermore, the risk of PED occurrence was found to be highest in the first tercile, showing a 7.89-fold higher risk compared to that of the third tercile for AMD without PED (95% CI: 2.87; 21.71, *p* < 0.001) and a 7.22-fold higher risk compared to that of the healthy controls (95% CI: 2.60; 20.06, *p* < 0.001). These findings suggest a strong association between the PRS and the risk of PED occurrence. The prevalence within the different groups is presented in Figure 2.

### 3.3. Rare Variant Burden Evaluation

We detected a total of 184 rare variants across 152 independent samples. Among the AMD patients with PED manifestation, we found 17 variants (36.2% of the 47 samples). In the AMD patients without PED, 86 variants were identified (45.3% of the 190 samples), while the control samples had 49 variants (50.5% of the 97 samples). The average numbers of variants per sample were 1.53, 1.38, and 1.3, respectively. The differences in numbers of patients with rare variants and the average numbers of variants per sample between the groups were not statistically significant. There was no statistical significance in predicted functional consequences or pathogenicity of the variants detected in the groups.

**Figure 3:**
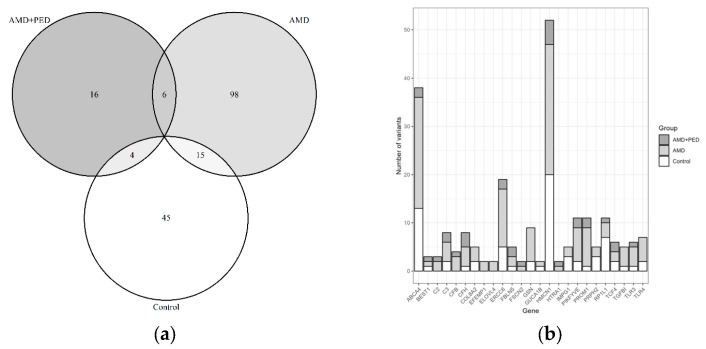
(**a**) Venn diagram showing the number of variants identified in patients within the tested groups; (**b**) distribution of variant occurrences in genes.

Notably, some variants were observed in two samples, including samples from different groups, as illustrated in Figure 3a. The genes with the highest numbers of variants were *HMCN1*, *ABCA4*, and *ERCC6*, as depicted in Figure 3b.

We found only two variants in the *ABCA4* gene in two patients in the AMD with PED group, while in the AMD group, there were 21 patients with 20 different variants, and in the control group, there were 13 different variants within 12 samples. The incidence of *ABCA4* variants in the AMD patients with PED was too low and the tested group was too small to test for significance; however, it might be of relevance to distinguish between AMD-only patients and those prone to PED development, and this should be investigated in a larger group of patients. There was no significant difference in either the functional effect or the pathogenicity of variants between the AMD without PED group and the control group. Additionally, there was no difference in number of samples with a variant in the AMD without PED group and the control group.

The situation was similar for the *HMCN1* gene. We found only two variants in the *HMCN1* gene in three patients in the AMD with PED group, while in the AMD group, there were 19 patients with 25 different variants, and in the control group, there were 17 different variants within 18 samples. The incidence of *HMCN1* variants in the AMD patients with PED was too low and the tested group was too small to test for significance; however, it might be of relevance to distinguish between AMD-only patients and those prone to PED development, and this should be investigated in a larger group of patients. There was no significant difference in either the functional effect or the pathogenicity of variants between the AMD without PED group and the control group. Moreover, there was no difference in number of samples with a rare variant in the AMD without PED group and the control group.

In two samples with developed PED, we found a three-variant pattern consisting of rs145108467 in *FBLN5* and co-occurrent *HMCN1* variants rs778062119 and rs747405345 with uncertain significance, although as single variants, they are considered to be likely benign. Each of these patients also bore one variant in the *TCF4* gene (rs777346668 or rs147445499). Similarly, two patients with AMD without PED had the same combination of *HMCN1* gene variants, rs114364265 and rs114629728, which were not described as co-occurrent in the gnomAD database. One of these patients had a third *HMCN1* variant (rs780673178), and for the second patient, we discovered additional variants in *COL8A2* (rs771963014) and *ABCA4* (rs62654395, described as pathogenic in autosomal recessive macular degeneration, such as Stargardt disease and cone–rod dystrophy, and this was submitted by Fulgent Genetics Laboratory as pathogenic in AMD). The list of identified variants is presented in Supplementary Table S1.

## 4. Discussion

### 4.1. PRS and Identified Variants

The findings of our study provide important insights into the genetic background of pigment epithelial detachment in age-related macular degeneration. By developing a polygenic risk score specific to AMD patients with pigment epithelial detachment, we managed to identify a significant association between the PRS and the risk of PED occurrence. This suggests that a substantial portion of the variability in PED susceptibility can be attributed to the cumulative effect of common genetic variants. The observed association between the PRS and PED risk aligns with the complex nature of age-related macular degeneration and its complications, which involve a combination of genetic and environmental factors. Previous studies have reported various prediction models for AMD by incorporating genetic variants alone or in combination with demographic, phenotypic, and/or environmental factors. For instance, Fritsche et al. presented a general genetic risk model of AMD based on seven loci with an AUC of 0.73 [29], while Hageman et al. validated the genetic model for developing a neovascular form of AMD with an AUC of 0.82 [11]. Our group previously presented a PRS model for AMD susceptibility in the Polish population based on targeted sequencing of 30 AMD-related genes, which had an AUC of 0.76 when considering age, sex, and smoking status as additional factors [16]. It has been noted that risk models incorporating lifestyle and demographic covariates tend to have higher predictive ability, as highlighted in a review by Cooke Bailey et al. [10].

Most models of susceptibility to AMD, its progression from the early to the late stages, and the determination of specific subtypes (CNV, GA) or treatment outcomes [30] involve recurrent gene loci, including *CFH*, *ARMS2/HTRA1*, *C2*, *C3,* and *CFB,* which is consistent with the GWAS results for AMD and related traits. One of those loci (*ARMS2*: A69S) was reported by Shijo et al., with the conclusion that the risk allele T was more frequently identified in Asian patients with drusenoid PED than in typical exudative AMD [31]. However, we did not find a similar correlation when the risk allele T frequency was compared between AMD patients without PED and patients manifesting PED. To date, no polygenic risk estimation specifically for PED complications in AMD has been reported. Our findings suggest that the genetic predisposition to PED is different from that of AMD without PED, thus confirming the need to study specific subtypes and manifestations within the AMD spectrum. We demonstrated a PRS approach that allowed the capture of the combined effect of six variants: rs760306 (*BEST1*), rs148662546 (*BEST1*), rs11569560 (*C3*), rs74600252 (*GUCA1B*), rs2240688 (*PROM1*), and rs185507582 (*TCF4*).

*BEST1*, also known as *VMD2*, is a gene that encodes the protein bestrophin, which functions as a chloride channel located in the basal membrane of the retinal pigment epithelium. This protein primarily serves as a chloride ion transporter from the RPE to the subretinal space. It plays a crucial role in maintaining the acid–base balance within RPE cells through pH regulation, which is essential for their proper functioning. Additionally, it regulates the intracellular levels of calcium ions (Ca^2+^) and plays a vital role in protecting RPE cells against oxidative stress, which can cause cell damage and disrupt retinal function. Mutations in the *BEST1* gene have been linked to bestrophinopathies, including Best disease, which exhibits symptoms overlapping with AMD, particularly in forms with late onset. Although the rs760306 variant included in our model did not show independent associations with AMD or PED in studies conducted by Kramer et al. and Allikments et al. [32,33], there is a possibility that rare bestrophin variants could contribute to an increased susceptibility to AMD. Allikments hypothesised that certain variants, even if they do not have a significant impact on the protein’s function, might still predispose individuals to developing AMD later in life [33]. The second variant in our model, rs148662546, has been associated with the ratio of omega-3 fatty acids to total fatty acids in GWASs [34]. However, its direct association with AMD and PED has not been evaluated. Nevertheless, its potential involvement in lipid deposits in the pathogenesis of AMD cannot be ruled out.

The *C3* gene encodes the complement component 3, a key component of the alternate immune pathway involved in the complement cascade. This gene plays a critical role in the complement system, which regulates immune responses and inflammation. Dysregulation of the complement cascade, including abnormalities in the complement component 3 protein, has been implicated in the pathogenesis of AMD. Several studies have investigated the association between genetic variations in the *C3* gene and the risk of developing AMD. For example, a study by Seddon et al. [35] reported a significant association between a common risk *C3* variant, rs2230199, and AMD. However, the intronic variant rs11569560 has not yet been documented in association with AMD or PED pathophysiology.

The *GUCA1B* gene encodes a calcium-binding protein that modulates the activity of guanylate cyclase in photoreceptor cells. Guanylate cyclase plays a crucial role in the phototransduction cascade by converting guanosine triphosphate (GTP) into cyclic guanosine monophosphate (cGMP), which is essential for the regulation of photoreceptor sensitivity. Differential expression of *GUCA1B* has been observed in comparisons between CNV retinas and normal or intermediate AMD retinas, suggesting its involvement in pathological choroidal neovascularization [36]. The exact mechanisms by which variations in the *GUCA1B* gene contribute to AMD and PED pathogenesis are not yet fully understood. However, it is believed that alterations in the *GUCA1B* protein may lead to dysregulation of guanylate cyclase activity, resulting in impaired phototransduction and compromised function of the RPE layer. In terms of retinal dystrophies, the molecular spectrum of *GUCA1B* has been evaluated, and several variants have been identified, although none of them have been classified as disease-causing [37]. The rs74600252 variant included in our model has not been previously published in association with AMD. Further research is needed to explore the potential link between this variant and AMD pathology.

The *PROM1* gene, also known as *CD133*, encodes a protein called prominin-1. Prominin-1 has an affinity for binding cholesterol in cholesterol-containing plasma membrane microdomains and, therefore, plays a role in organising the apical plasma membrane in epithelial cells, including RPE. During early retinal development, it acts as a crucial regulator of disk morphogenesis and, as animal models show, the loss of function leads to photoreceptor degeneration [38,39]. Mutations in this gene have been identified as the underlying cause of retinitis pigmentosa and Stargardt disease [40]. The protein is commonly expressed in adult stem cells, where it is believed to play a role in preserving stem cell properties by suppressing differentiation. The expression of *PROM1* is also associated with various types of cancer and has been extensively studied as a stem cell marker and early biomarker for lung, gastric, and breast cancer [41,42,43]. Moreover, the rs2240688 variant, which is located in a miRNA binding site, has been analysed in terms of the clinical outcome of anti-VEGF bevamizumab-based cancer treatment [44]. Since bevamizumab is also used in the treatment of CNV, it is worth investigating whether this variant or PRS model can predict treatment outcomes in AMD patients with or without PED.

The *TCF4* gene, also known as Transcription Factor 4, is involved in various cellular processes, including gene transcription and regulation of cell growth and differentiation. While variants in the *TCF4* gene have been associated with neurological disorders, such as schizophrenia, and bipolar disorder, in GWASs, one has been specifically linked to Fuchs endothelial corneal dystrophy (FECD). It has been demonstrated that excessive trinucleotide repetition causing FECD is not observed in AMD patients [45]. To date, no associations between *TCF4* variants and AMD or PED have been published. However, it is important to note that research in this area is ongoing, and further investigations may provide more insights into the potential involvement of *TCF4* in the pathogenesis of AMD and PED.

The PRS demonstrated a strong predictive value, with the first tercile of the PRS showing a significantly higher risk of PED compared to the third tercile. This highlights the potential clinical utility of the PRS as a risk stratification tool in AMD patients, aiding in early detection and personalised management strategies. The identification of individuals at higher risk for PED may facilitate targeted interventions and monitoring to prevent or delay disease progression.

### 4.2. Rare Variant Burden

In addition to analysing common variants, we also conducted investigations into the role of rare variants in selected candidate genes associated with AMD, including those related to PED. However, our analysis did not uncover any significant associations between rare variants and PED in the AMD cohort. This may be due to the limited sample size and the inherent rarity of these variants within the population. Therefore, further studies with larger cohorts and functional investigations are necessary to thoroughly assess the contributions of rare variants to PED pathogenesis. Nevertheless, we did make several incidental findings during our research. In one patient who carried the p.Gly863Ala variant in the *ABCA4* gene, we also detected a commonly reported hypomorphic variant, p.Asn1868Ile, within the same gene. Similarly, in another patient, we identified the hypomorphic variant p.Asn1868Ile along with the pathogenic variant p.Asn96Asp in *ABCA4*. The rare variants p.Asn1868Ile and p.Gly863Ala have been previously observed in Polish patients with inherited retinal diseases, while p.Asn96Asp has not been reported before [46]. These findings highlight the importance of further investigation to determine whether the observed degeneration is caused by AMD or possibly a late-onset mild *ABCA4* disease with central atrophy [47,48].

It is worth noting that our study focused on Polish patients, providing valuable insights into the genetic background of PED in this population. However, genetic variations can differ across ethnicities, and further studies in diverse populations are needed to validate our findings and expand our understanding of the genetic architecture of PED in AMD. While our study primarily focused on the genetic aspects of PED in AMD, it is important to acknowledge that environmental and lifestyle factors also play a significant role in disease development and progression. Factors such as smoking, diet, and sunlight exposure have been implicated in AMD pathogenesis, and their interaction with genetic factors should be considered in future studies.

## 5. Conclusions

If the development of PED is related to the individual PRS result, which requires further confirmation, this may shed new light on the pathogenesis of AMD. Despite the unequivocal genetic background of the disease, the results of the search for single variants responsible for the disease phenotype are not encouraging. Our results suggest that a shift in approach to analysing gene groups rather than single variants may be associated with the accumulation of newly identified factors influencing the phenotype.

## Figures and Tables

**Table 1:** Variants included in the PRS model. Nucleotide numbering according to HGVS nomenclature.

dbSNP ID	Gene	Coding Position (hg19)	Type	Germline Classification	Allele Frequency—All Populations	Allele Frequency—Non-Finnish European
rs760306	*BEST1*	NM_004183.4:c.482-24C > A	intronic	likely benign	0.4039	0.2605
rs148662546	*BEST1*	NM_004183.4:c.868-17C > A	intronic	benign	0.004014	0.002502
rs11569560	*C3*	NM_000064.4:c.4457-71C > T	intronic	benign	0.03460	0.03977
rs74600252	*GUCA1B*	NM_002098.6:c.208-81A > T	intronic	benign	0.05707	0.07103
rs2240688	*PROM1*	NM_006017.3:c.*667A > C	3′UTR	benign	0.2316	0.2877
rs185507582	*TCF4*	NM_001083962.2:c.*5-68T > A	intronic	benign	0.02135	0.03303

## Data Availability

Summary statistics of AMD GWA studies were downloaded from the NHGRI-EBI GWAS Catalog, study accession GCST90086108 http://ebi.ac.uk (accessed on 19 May 2023). The target data can be obtained from NeuStemGen STRATEGMED consortium members upon request.

## Supplementary Materials

The following supporting information can be downloaded at: https://www.mdpi.com/article/10.3390/genes14091707/s1, Table S1: Rare variants within coding sequences of enriched genes identified in, at most, two patients.
